# MEETINGS CALENDAR - 2017

**Published:** 2016

## January

### ►15 to 20 The 35^th^ Annual International Symposium: Clinical update in Anesthesiology, Surgery and Perioperative Medicine

Cancun, Mexico

Informations: George Silvay

Phone: 212 241 8346

Fax: 212 876 2009

E-mail: george.silvay@mountsinai.org

Site: www.clinicalupdateinanesthesiology.org

### ►19 Cardiac Tumour Conference - Cardiac Endocrine Tumours

Toronto, Canada

Informations: Continuing Professional Development, University of Toronto

Phone: (416) 978-2719

E-mail: info-sur1785@cpdtoronto.ca

Site: www.cpd.utoronto.ca/cardiactumours

### ►21 to 25 STS 53rd Annual Meeting and STS/AATS Tech-Con 2017

Houston, United States

Informations: The Society of Thoracic Surgeons

E-mail: education@sts.org

Site: www.sts.org/annualmeeting

### ►22 to 24 2017 STS and CTSNet Career Fair

Houston, United States

Informations: Jim Cook

Phone: (513) 451-7597

E-mail: jcook@yourmembership.com

## February

### ►5 to 7 Mitral Valve Meeting 2017

Zurich, Switzerland

Informations: Judith Kutnjak

Phone: +43 1 867 49 44 22

E-mail: judith.kutnjak@ee-hsec.org

Site: http://heart-team.org/2017-Mitral_Valve_Meeting/index.php

### ►8 and 9 8th Advanced VATS Course

Leeds, United Kingdom

Informations: Linda Caton

Phone: +44 11320 68760

E-mail: linda.caton@nhs.net

Site: http://www.vatscourse.com/

### ►22 to 24 Hands-on Cardiac Morphology

London, United Kingdom

Informations: Amy

Phone: 7025085200

Fax: 02073518544

E-mail: morphology@rbht.nhs.uk

Site: www.rbht.nhs.uk/cardiacmorphology

